# Differential Prognostic Impact of Risk-Prediction Models for Heart Failure in Acute Myocardial Infarction: The Original and Revised Heart Failure Time-Points

**DOI:** 10.3390/jcm13092501

**Published:** 2024-04-24

**Authors:** Kazunari Asada, Yuichi Saito, Hiroki Goto, Hiroaki Yaginuma, Takanori Sato, Osamu Hashimoto, Hideki Kitahara, Yoshio Kobayashi

**Affiliations:** 1 Department of Cardiovascular Medicine, Chiba University Graduate School of Medicine, Chiba 260-8670, Japan; asdkznr123@gmail.com (K.A.); moritap77@gmail.com (H.G.); hiroaki19940218baseball@gmail.com (H.Y.); t-satoh@outlook.jp (T.S.); hidekita.0306@gmail.com (H.K.); yuiryosuke@msn.com (Y.K.); 2 Department of Cardiology, Chiba Emergency and Psychiatric Medical Center, Chiba 261-0024, Japan; osamu112358@gmail.com

**Keywords:** myocardial infarction, heart failure, risk score

## Abstract

**Background:** We previously developed a risk-scoring system for heart failure (HF) in patients with acute myocardial infarction (MI), namely “HF time-points (HFTPs)”. In the original HFTPs, the presence of HF on admission, during hospitalization, and at short-term follow-up was individually scored. This study examined whether the revised HFTPs, with additional scoring of previous HF, provide better predictivity. **Methods:** This multicenter registry included a total of 1331 patients with acute MI undergoing percutaneous coronary intervention. HF was evaluated at four time-points before and after acute MI onset: (1) a history of HF; (2) elevated natriuretic peptide levels on admission; (3) in-hospital HF events; and (4) elevated natriuretic peptide levels at a median of 31 days after the onset. When HF was present at each time-point, one point was assigned to a risk scoring system, namely the original and revised HFTPs, ranging from 0 to 3 and from 0 to 4. The primary endpoint was a composite of cardiovascular death and HF rehospitalization after discharge. **Results:** Of the 1331 patients, 65 (4.9%) had the primary outcome events during a median follow-up period of 507 (interquartile range, 335–1106) days. The increase in both original and revised HFTPs was associated with an increased risk of the primary outcomes in a stepwise fashion with similar diagnostic ability. **Conclusions:** The original and revised HFTPs were both predictive of long-term HF-related outcomes in patients with acute MI undergoing percutaneous coronary intervention. Yet, the original HFTPs may be sufficient to estimate HF risks after MI.

## 1. Introduction

Ischemic heart disease, including myocardial infarction (MI), is a common etiology of heart failure (HF) across the world, accounting for approximately 50% in the current era [1,2], and the presence of HF is a strong predictor of mortality in patients with acute MI [3]. HF developing after MI is a consequence of cardiomyocyte death and scar formation. Left ventricular remodeling post-MI includes mechanical (pressure and volume overload) and non-mechanical (neurohumoral activation of renin-angiotensin-aldosterone and sympathetic nervous systems, activation of pro-inflammatory pathways, and changes in the extracellular matrix) pathophysiology, leading to impaired systolic and diastolic function [4]. It is well known that the presence of worse HF conditions (i.e., higher Killip class) after acute MI is associated with higher short-term mortality in a stepwise manner [5], and an administrative database study demonstrated that new-onset HF after acute MI occurred in one out of every three patients and denoted an “exceptionally” high long-term mortality rate [6]. To identify patients with HF and stratify risks of adverse outcomes, the evaluation of natriuretic peptides (NPs), such as B-type natriuretic peptide (BNP) and N-terminal pro-BNP (NT-proBNP), is recommended in patients with acute MI in the international guidelines [7,8]. NPs are produced by ventricular cardiomyocytes and released into circulation in response to volume and pressure overload to the ventricular wall [9]. In daily clinical practice, elevated levels of NPs are reportedly associated with poor outcomes after acute MI. For instance, a previous study found that the level of BNP on admission was an independent predictor of all-cause mortality at 30 days in patients with acute MI [10]. Similarly, a Japanese study demonstrated that BNP levels 3 to 4 weeks after acute MI were independently associated with the risk of cardiac death at a mean follow-up of 58.6 months [11]. In addition to the elevated NP levels, clinical HF events are also related to worse clinical outcomes after MI [3,12]. Taken together, a comprehensive risk assessment incorporating both NP levels and clinical evidence of HF events may contribute to better risk prediction in patients with acute MI. Given that an intensive therapeutic approach of rapid up-titration of guideline-directed medical therapy resulted in better outcomes in hospitalized HF patients, as shown in a recent randomized trial [13], timely risk stratification is clinically important, and subsequent medical intervention may benefit patients with acute MI.

In this context, we previously developed a risk-predicting model, namely “HF time-points (HFTPs)”, showing that HF evaluation at different time-points provided diagnostic value for future clinical events in a setting of acute MI [14]. In our previous study, patients with acute MI were assessed as to whether HF was present at three different time-points: (1) high NP levels on admission; (2) in-hospital HF events; and (3) high NP levels at short-term follow-up [15]. With the increase in the number of patients with HF status at the time-points, the rate of all-cause death and HF progressively increased after acute MI [14]. In the original HFTPs, however, a history of HF before admission for MI was not included due to the limited sample size, which also has a prognostic value in patients with acute MI in previous studies [16,17]. Therefore, in the present study, we added a history of HF besides the original criteria to develop the revised HFTPs and evaluated its diagnostic ability for HF events after acute MI.

## 2. Materials and Methods

### 2.1. Study Population

This was a series of retrospective, observational, multicenter studies [18,19,20]. From January 2012 to March 2021, a total of 2485 patients with acute MI underwent percutaneous coronary intervention (PCI) at four hospitals (Chiba University Hospital, Eastern Chiba Medical Center, Chiba Emergency and Psychiatric Medical Center, and Chiba Aoba Municipal Hospital). In the present study, acute MI included ST-segment elevation and non-ST-segment elevation MI, defined according to the fourth universal definition [21]. PCI procedures were performed based on the local standard practice with the predominant use of dual antiplatelet therapy, radial access, intracoronary imaging, new-generation drug-eluting stents, and mechanical circulatory support devices [22,23,24,25,26,27]. Patients with in-hospital onset acute MI (*n* = 24), late presentation >48 h (*n* = 81), in-hospital death (*n* = 209), missing data on NP levels on admission or at short-term follow-up (*n* = 744), and no follow-up information after hospital discharge (*n* = 96) were hierarchically excluded. Thus, a total of 1331 patients were included in the present study. Cardiovascular risk factors such as hypertension, diabetes, dyslipidemia, and current smoking were defined based on the Japanese Association of Cardiovascular Intervention and Therapeutics criteria [28]. Hypertension was defined as a previous diagnosis of hypertension or previous antihypertensive medications, or newly diagnosed hypertension during hospitalization with systolic blood pressure ≥140 mm Hg and/or diastolic blood pressure ≥90 mm Hg. Diabetes was defined as a previous diagnosis of diabetes or the previous use of glucose-lowering drugs, or a level of hemoglobin A1c ≥6.5%. Dyslipidemia was defined as having low-density lipoprotein cholesterol ≥140 mg/dL, high-density lipoprotein cholesterol <40 mg/dL, or fasting triglycerides >150 mg/dL, or a previous diagnosis of dyslipidemia. Low- and high-density lipoprotein cholesterol levels were assessed in either a fasting or non-fasting state. Other blood examination findings, including hemoglobin and creatinine, were also assessed. Additionally, patients having a history of smoking within the past year were defined as being current smokers. Chronic kidney disease was defined based on an estimated glomerular filtration rate <60 mL/min/1.73 m^2^. Cardiogenic shock was defined as a sustained episode of systolic blood pressure <80 mm Hg and/or a cardiac index <1.8 L/min/m^2^ (regardless of the measurement method) despite the maximum treatment determined to be secondary to cardiac dysfunction and/or the need for parenteral inotropic or vasopressor agents or mechanical support, including an intra-aortic balloon pump to maintain blood pressure and cardiac index above specified levels within 24 h prior to the initiation of PCI [28]. This study was conducted in accordance with the Declaration of Helsinki and was centrally approved by the ethics committee of Chiba University Hospital. Informed consent for the present study was obtained in an opt-out manner. 

### 2.2. Heart Failure Evaluation

**Figure 1:**
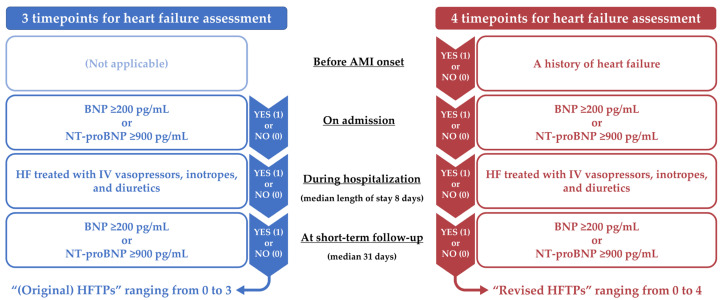
Time-points of heart failure assessment in the original and revised scores. AMI, acute myocardial infarction; BNP, B-type natriuretic peptide; HF, heart failure; HFTPs, heart failure time-points; IV, intravenous; NT-proBNP, N-terminal pro-B-type natriuretic peptide.

In the present study, HF status was evaluated at four different time-points: (1) before admission; (2) on admission; (3) during hospitalization; and (4) at short-term follow-up (Figure 1). Levels of BNP or NT-proBNP were measured on admission and at short-term follow-up in a real-world setting. The short-term follow-up NP level was obtained immediately before discharge or at the 1-month visit in routine clinical practice at the four institutions [14]. Patients were considered to have HF when NP levels on admission and at short-term follow-up were high, with BNP ≥200 pg/mL or NT-proBNP ≥ 900 pg/mL, according to the guidelines [29]. A history of HF before admission was assessed based on the medical record. In-hospital HF was defined as the use of intravenous diuretics (e.g., furosemide) and vasopressors or inotropes (e.g., norepinephrine and dobutamine) [30]. The use of intravenous vasodilators such as nitrates and nicorandil did not fulfill the definition of in-hospital HF. The original HFTPs were evaluated using the criteria at the three time-points: on admission, during hospitalization, and at short-term follow-up [14], while the revised HFTPs in the current study were assessed with HF status at the four time-points, including previous HF before admission (Figure 1). When HF was present at each time-point, one point was added to the risk-scoring models. Thus, the original and revised HFTPs ranged from 0 to 3 and from 0 to 4, respectively. 

### 2.3. Outcomes and Statistical Analysis

Follow-up data were ascertained from medical records at individual institutions. The primary outcome of the present study included cardiovascular death and HF rehospitalization after discharge. Cardiovascular death was defined as death presumably resulting from cardiovascular causes, according to the Academic Research Consortium-2 consensus document [31]. Cardiovascular death included death caused by acute MI, stroke, and cardiovascular procedures and resulting from HF and cardiovascular hemorrhage. Sudden cardiac—including unwitnessed—death and death from other cardiovascular causes were also included as cardiovascular death [31]. 

Statistical analysis was conducted using EZR version 1.61 (Saitama Medical Center, Jichi Medical University, Saitama, Japan), which is a graphical user interface for R (The R Foundation for Statistical Computing, Vienna, Austria). Data are expressed as mean ± standard deviation, median [interquartile range], or frequencies with percentages, as appropriate. Continuous variables were assessed with Student’s *t*-test, and categorical variables were compared using Fisher’s exact test. The time to the primary outcome events (cardiovascular death and HF rehospitalization after discharge) was estimated using the Kaplan–Meier method with the log-rank test. The date of discharge was set as a landmark. The receiver operating characteristics (ROC) curve analysis was performed based on the primary outcomes. In the ROC curve analysis, the best cut-off value was established by finding the values that corresponded to the maximum average sensitivity and specificity. The area under the curve (AUC) of the ROC curve was compared with the Delong method. Multivariable analysis was performed using a Cox proportional hazards model for the primary outcomes, with factors included in the model in our previous report [14]. A *p* value < 0.05 was considered statistically significant.

## 3. Results

A total of 1331 patients with acute MI who underwent PCI and survived to discharge were included in the present analysis. Overall, the mean age was 67.1 ± 12.2 years, and 77.8% were men (Table 1). Cardiovascular risk factors were commonly found, with hypertension in 68.3%, diabetes in 34.6%, dyslipidemia in 66.9%, and current smoking in 37.3%, respectively. The majority of patients suffered from ST-segment elevation MI (72.4%), and cardiogenic shock was found in 9.2% (Table 1). The median length of hospital stay, duration of short-term follow-up for NP measurement, and follow-up period after discharge were 8 [6, 14], 31 [19, 39], and 507 [355, 1106] days, respectively. During the follow-up period, 65 (4.9%) patients experienced the primary outcomes, including cardiovascular death and HF rehospitalization after discharge. No patients had the primary outcome events before the short-term follow-up. 

Table 1 shows baseline characteristics between patients with and without adverse events. Patients with the primary outcome events had older age, anemia, and impaired left ventricular ejection fraction and renal function as compared to those without (Table 1). The prevalence of HF was significantly higher at all four time-points in patients with the primary outcome events than in their counterparts before admission (7.7% vs. 1.7%, *p* = 0.007), on admission (60.0% vs. 24.6%, *p* < 0.001), during hospitalization (61.5% vs. 19.6%, *p* < 0.001), and at short-term follow-up (73.8% vs. 33.1%, *p* < 0.001) (Table 1). Overall, the median original and revised HFTPs were 0 [0, 2]. 

**Figure 2:**
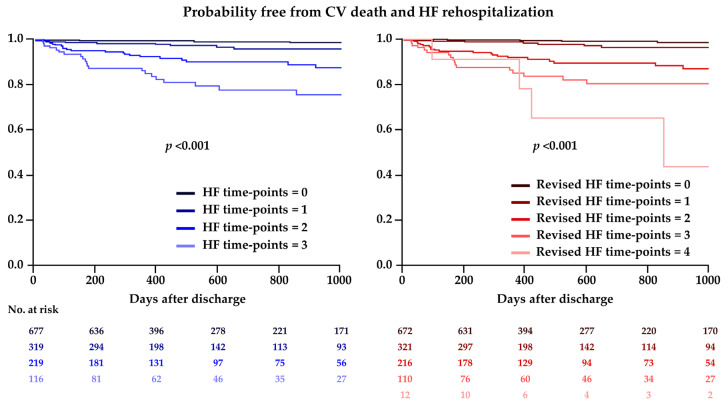
Kaplan–Meier analysis for the primary endpoint after discharge according to the heart failure time-points; CV, cardiovascular; HF, heart failure.

**Figure 3:**
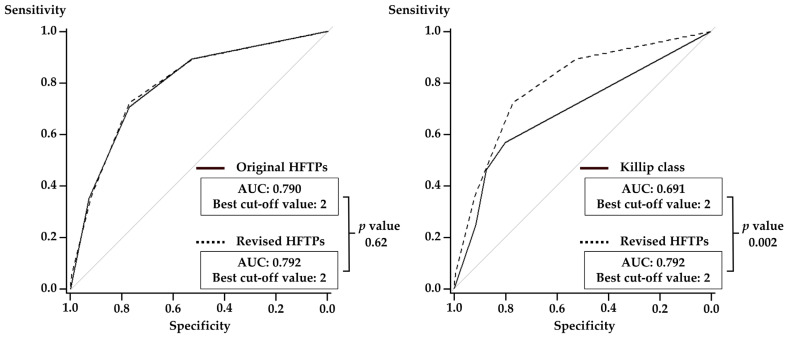
Receiver operating characteristic curve analysis for the primary outcomes between revised HFTPs and original HFTPs (**left**) or Killip class (**right**). AUC, area under the curve; HFTPs, heart failure time-points.

Kaplan–Meier analysis demonstrated that the higher original and revised HFTPs were associated with an increased risk of cardiovascular death and HF rehospitalization after discharge in a stepwise manner (Figure 2). In the ROC curve analysis, no significant difference in a prognostic value for the primary endpoint was observed between the original and revised HFTPs (AUC 0.79 vs. 0.79, *p* = 0.62), while the revised HFTPs had a significantly greater AUC as compared with Killip classification (Figure 3). With the best cut-off values for predicting cardiovascular death and HF rehospitalization after discharge, sensitivity, specificity, positive predictive value, negative predictive value, and accuracy of the original and revised HFTPs were 70.8%, 77.2%, 13.7%, 98.1%, and 76.9% and 72.3%, 77.0%, 13.9%, 98.2%, and 76.8%. Multivariable analysis indicated that the original HFTPs of 2 and 3 and the revised HFTPs of ≥2 were independently associated with the occurrence of primary outcome events after discharge, in addition to anemia and impaired renal function, while one point was related with marginal significance in the multivariable models in both scores (Table 2).

## 4. Discussion

In the present multicenter registry study, we evaluated the prognostic impact of revised HFTPs, a risk-scoring system based on HF status at different time-points, in patients with acute MI undergoing PCI. The original HFTPs consist of the presence or absence of HF on admission, during hospitalization, and at short-term follow-up, while the revised HFTPs further incorporate a history of HF before MI. Although the presence of previous HF was associated with an increased risk of cardiovascular death and HF rehospitalization after discharge, the addition of previous HF as a part of HFTP criteria did not result in better risk stratification. Their similar diagnostic abilities may support the use of both original and revised HFTPs for estimating patient risks of long-term death and HF events after MI. 

HF developing after acute MI is common with the underlying mechanisms of cardiac remodeling and systolic and diastolic dysfunction, which is a powerful predictor of death, while the presence of HF has important clinical implications for treatment [4,15,32]. It has been established that the presence of HF is associated with worse clinical outcomes in patients with acute MI. In a historical study, the higher Killip class was associated with an increased mortality rate at 30 days after acute MI: 5.1% in those with Killip class 1, 13.6% in class 2, 32.2% in class 3, and 57.8% in class 4, respectively [5]. The prognostic impact of the Killip class was also demonstrated in patients with MI with non-obstructive coronary artery disease [33]. For long-term outcomes, a recent study using a Medicare database showed that the presence of new-onset HF after acute MI was significantly associated with higher long-term mortality: 68.7% at 5 years in patients with new-onset HF and 38.4% in those without [6]. Previous investigations have also found that HF at several time-points is related to the prognosis in patients with acute MI. A large-scale administrative database study indicated that among hospitalized acute MI patients, pre-existing HF was a predictor of in-hospital mortality [17]. A sub-analysis of the ENTIRE-TIMI-23 (Enoxaparin Tenecteplase-Tissue-Type Plasminogen Activator With or Without Glycoprotein IIb/IIIa Inhibitor as Reperfusion Strategy in ST-Segment Elevation Myocardial Infarction-Thrombolysis In Myocardial Infarction-23) trial demonstrated that an elevated level of BNP on admission with a cut-off value of 80 pg/mL was associated with a seven-fold higher 30-day mortality risk [10]. In-hospital HF events after acute MI are also known as predictors of poor prognosis [12]. When assessed 3 to 4 weeks after acute MI, a higher BNP level was identified as a significant factor related to higher long-term cardiac mortality, with the best cut-off value of 180 pg/mL [11]. In summary, the presence of HF at different times—before and after acute MI—is a strong prognostic marker, and thus, it may be reasonable to stratify patient risks, particularly for HF-related events, based on HF status at such time-points. 

In this context, we developed the original HFTPs in a previous report [14]. In the original study, we included a total of 600 patients with acute MI undergoing PCI at two tertiary referral centers. With the same criteria for HF evaluation as was employed in the present study on admission (BNP ≥ 200 pg/mL or NT-proBNP ≥ 900 pg/mL), during hospitalization (clinical HF events), and at short-term follow-up (BNP ≥ 200 pg/mL or NT-proBNP ≥ 900 pg/mL), the original HFTPs predicted composite endpoints of all-cause death and HF rehospitalization [14]. Although patients with the higher original HFTPs were likely to be older and have impaired renal function and anemia, multivariable analysis identified the original HFTPs as a predictor of the clinical events, particularly when the score was 2 or 3 [14]. As shown previously, the present study confirmed that the original HFTPs were predictive of long-term HF-related events in patients with acute MI [14]. For further improvement in predictivity, we hypothesized that the addition of HF evaluation at different time-points may be efficient. In the pivotal GRACE (Global Registry of Acute Coronary Events) study, nine variables, such as older age, higher heart rate, lower blood pressure, ST-segment deviation on electrocardiogram, renal impairment, cardiogenic shock, and cardiac arrest, were identified to be associated with a composite of death and MI at six months in patients presenting with acute coronary syndrome, of which the presence of previous HF was also included [16]. In addition, a recent large-scale administrative database study demonstrated that patients with pre-existing HF had higher mortality than those without, irrespective of their left ventricular ejection fraction [17]. However, because the endpoints of these studies did not include HF-related events, the prognostic impact of previous HF before MI on the development of HF after MI during long-term follow-up remains unclear. In the present study, the univariable analysis showed that a history of HF was more frequently observed in patients with cardiovascular death and HF rehospitalization than in those without (1.7% vs. 7.7%, *p* = 0.007), suggesting that the presence of previous HF was at least an indicator of future HF events after MI. Nonetheless, the addition of HF before MI in the revised HFTPs did not lead to improved diagnostic ability as compared to the original score, possibly explained by the small sample size of patients with previous HF (i.e., 2.0%) in this population. Therefore, the original HFTPs may be sufficient for predicting HF-related events after acute MI. Another important point to note in this study was that one point in the original and revised HFTPs was indicative of adverse outcomes, significantly in univariable analysis and with marginal significance in multivariable analysis. This signal was not found in our previous report, in which the sample size was smaller, and the primary endpoint included all-cause rather than cardiovascular death [14]. Thus, beyond the fact that the risk of cardiovascular death and HF was progressively increased with the increase in HFTPs, the presence of HF, even at one time-point, may be taken into account when treating and managing patients with acute MI. 

The early and accurate risk stratification may improve HF-related clinical outcomes in patients with acute MI, owing to intensified medical intervention with anti-HF drugs. The STRONG-HF (Safety, Tolerability and Efficacy of Rapid Optimization, Helped by NT-proBNP Testing, of Heart Failure Therapies) trial enrolled hospitalized patients with acute HF, irrespective of left ventricular ejection fraction, and evaluated the effect of an intensive treatment strategy of rapid up-titration of guideline-directed HF medication on clinical outcomes as compared to the control treatment, resulting in lower adverse event rates in the experimental arm at 180 days [13]. The randomized trial did not include patients with acute MI (one of the exclusion criteria) but supported the therapeutic potential of the intensive treatment strategy to reduce HF events. In the randomized SAVE (Survival and Ventricular Enlargement) trial, early initiation of angiotensin-converting enzyme inhibitor (captopril) within 3 to 16 days after acute MI was associated with better survival at the mean of 42 months [34]. Similarly, the beneficial effect of early initiation of β-blocker and mineralocorticoid receptor antagonist in a setting of acute MI complicated by reduced left ventricular ejection fraction has also been established in pivotal clinical studies, such as the CAPRICORN (Carvedilol Post-Infarct Survival Control in Left Ventricular Dysfunction) and EPHESUS (Eplerenone Post-AMI Heart Failure Efficacy and Survival Study) trials [35,36]. More recently, the safety and possible therapeutic effect of angiotensin receptor-neprilysin inhibitor and sodium-glucose cotransporter-2 have been suggested in the EMMY (Empagliflozin in Myocardial Infarction) and PARADISE-MI (Prospective ARNI vs. ACE inhibitor trial to DetermIne Superiority in reducing heart failure Events after Myocardial Infarction) trials [37,38]. Although not established yet, early intensive medical treatment with anti-HF drugs in high-risk patients may be beneficial after acute MI. Further studies are warranted to evaluate whether risk-based intervention is effective in reducing HF-related events in a setting of acute MI. Prospective studies are needed to confirm our results and to show the clinical effectiveness of HFTP-based therapeutic approaches. 

There are some limitations in the present study. This was a retrospective study in which a proportion of patients were excluded mainly due to the lack of NP data. Data on some comorbidities, including chronic obstructive pulmonary disease, were missing. Additionally, baseline characteristics, including medications (e.g., aspirin and statin), significantly differed between patients with and without adverse events, suggesting the presence of selection bias in a real-world clinical setting. Even with the increased sample size in the present study compared to our previous report [14], the number of HF events at each time-point was relatively small. Although the efficacy of sodium-glucose co-transporter-2 inhibitors for HF has been established [39], the number of patients on the drug was also small. In our original study, the primary endpoint was a composite of all-cause death and HF rehospitalization, while in the present study, it was set as cardiovascular death and HF rehospitalization. Because either BNP or NT-proBNP was employed in the participating study centers, NP levels were used as being dichotomous with the thresholds (i.e., BNP of 200 pg/mL and NT-proBNP of 900 pg/mL). In addition, we believe that external validation of HFTPs with different study cohorts and investigators is needed. 

## 5. Conclusions

In patients with acute MI who underwent PCI and survived to discharge, the evaluation of HF status at four different time-points, including a history of HF, an elevated NP level on admission, in-hospital HF events, and a higher NP level at short-term follow-up, was useful to stratify the risk of cardiovascular death and HF rehospitalization after discharge, although the addition of the presence of previous HF as a part of the scoring model did not provide incremental improvement in the prognostic impact.

## Figures and Tables

**Table 1:** Baseline characteristics.

Variable	All (*n* = 1331)	Adverse Event (−) (*n* = 1266)	Adverse Event (+) (*n* = 65)	*p* Value
Age (years)	67.1 ± 12.2	66.9 ± 12.3	70.9 ± 11.0	0.009
Men	1036 (77.8%)	984 (77.7%)	52 (80.0%)	0.76
Body mass index (kg/m^2^)	24.5 ± 3.9	24.6 ± 3.9	23.4 ± 4.2	0.01
Hypertension	909 (68.3%)	856 (67.6%)	53 (81.5%)	0.02
Diabetes	461 (34.6%)	436 (34.4%)	25 (38.5%)	0.51
Dyslipidemia	890 (66.9%)	850 (67.1%)	40 (61.5%)	0.35
Current smoker	496 (37.3%)	474 (37.4%)	22 (33.8%)	0.60
Previous MI	105 (7.9%)	94 (7.4%)	11 (16.9%)	0.02
Previous PCI	142 (10.7%)	128 (10.1%)	14 (21.5%)	0.007
eGFR (mL/min/1.73 m^2^)	66.7 ± 23.1	67.7 ± 22.6	46.9 ± 23.7	<0.001
Hemoglobin (g/dL)	14.2 ± 2.0	14.2 ± 1.9	12.8 ± 2.3	<0.001
LVEF (%)	47.6 ± 11.2	48.0 ± 11.0	40.6 ± 13.0	<0.001
Cardiogenic shock	123 (9.2%)	119 (9.4%)	4 (6.2%)	0.51
Type of MI				0.26
STEMI	964 (72.4%)	921 (72.7%)	43 (66.2%)	
NSTEMI	367 (27.6%)	345 (27.3%)	22 (33.8%)	
Medication at discharge				
Aspirin	1227 (92.2%)	1172 (92.6%)	55 (84.6%)	0.03
P2Y12 inhibitor	1283 (96.3%)	1225 (96.8%)	60 (92.3%)	0.07
Oral anticoagulation	191 (14.4%)	174 (13.7%)	17 (26.2%)	0.01
Statin	1267 (95.2%)	1210 (95.6%)	57 (87.7%)	0.01
ACE-i or ARB	1043 (78.4%)	992 (78.4%)	51 (78.5%)	1.00
β-blocker	1064 (79.9%)	1011 (79.9%)	53 (81.5%)	0.87
MRA	249 (18.7%)	229 (18.1%)	20 (30.8%)	0.01
Diuretic	265 (19.9%)	227 (17.9%)	38 (58.5%)	<0.001
HF history before admission	26 (2.0%)	21 (1.7%)	5 (7.7%)	0.007
High NP on admission	350 (26.3%)	311 (24.6%)	39 (60.0%)	<0.001
In-hospital HF	288 (21.6%)	248 (19.6%)	40 (61.5%)	<0.001
High NP at follow-up	467 (35.1%)	419 (33.1%)	48 (73.8%)	<0.001

Adverse event indicates the primary outcome, a composite of cardiovascular death and HF rehospitalization after discharge. In-hospital HF was defined as the use of intravenous diuretics (e.g., furosemide) and vasopressors or inotropes (e.g., norepinephrine and dobutamine) during the index hospitalization for acute MI. ACE-i, angiotensin-converting enzyme inhibitor; ARB, angiotensin II receptor blocker; eGFR, estimated glomerular filtration rate; HF, heart failure; LVEF, left ventricular ejection fraction; MI, myocardial infarction; MRA, mineralocorticoid receptor antagonist; NP, natriuretic peptide; NSTEMI, non-ST-segment elevation myocardial infarction; PCI, percutaneous coronary intervention; STEMI, ST-segment elevation myocardial infarction.

**Table 2:** Factors associated with primary endpoint.

Variable	Univariable		Multivariable (Original HFTPs)		Multivariable (Revised HFTPs)	
	HR (95% CI)	*p* Value	HR (95% CI)	*p* Value	HR (95% CI)	*p* Value
Age (years)	1.04 (1.01–1.06)	0.002	1.00 (0.98–1.03)	0.84	1.00 (0.98–1.03)	0.86
Men	1.10 (0.60–2.01)	0.77	1.60 (0.84–3.05)	0.15	1.57 (0.82–3.01)	0.17
BMI (kg/m^2^)	0.91 (0.86–0.98)	0.009	0.96 (0.89–1.04)	0.30	0.96 (0.89–1.04)	0.32
Hypertension	2.04 (1.09–3.82)	0.03	1.45 (0.73–2.84)	0.28	1.44 (0.73–2.83)	0.29
eGFR (mL/min/1.73 m^2^)	0.96 (0.95–0.97)	<0.001	0.98 (0.97–0.99)	0.002	0.98 (0.97–0.99)	0.004
Hemoglobin (g/dL)	0.75 (0.68–0.83)	<0.001	0.87 (0.76–0.99)	0.04	0.87 (0.76–0.99)	0.04
LVEF (%)	0.94 (0.92–0.96)	<0.001	0.98 (0.95–1.00)	0.07	0.98 (0.95–1.00)	0.07
Cardiogenic shock	0.65 (0.24–1.80)	0.41	0.36 (0.11–1.19)	0.10	0.38 (0.12–1.24)	0.11
Original HFTPs						
0	Reference		Reference			
1	3.45 (1.36–8.76)	0.009	2.72 (0.99–7.45)	0.051		
2	10.54 (4.52–24.6)	<0.001	6.29 (2.35–16.8)	<0.001		
3	21.48 (9.21–50.1)	<0.001	7.37 (2.48–21.9)	<0.001		
Revised HFTPs						
0	Reference				Reference	
1	3.11 (1.21–8.03)	0.02			2.48 (0.89–6.90)	0.08
2	11.19 (4.82–26.0)	<0.001			6.59 (2.47–17.6)	<0.001
3	18.08 (7.60–43.0)	<0.001			6.65 (2.22–19.9)	<0.001
4	40.32 (11.8–138.0)	<0.001			9.70 (2.25–41.8)	0.002

BMI, body mass index; CI, confidence interval; eGFR, estimated glomerular filtration rate; HFTPs, heart failure time-points; HR, hazard ratio; LVEF, left ventricular ejection fraction.

## Data Availability

The data from this study are available upon reasonable request.
